# Atg7- and Keap1-dependent autophagy protects breast cancer cell lines against mitoquinone-induced oxidative stress

**DOI:** 10.18632/oncotarget.1715

**Published:** 2014-03-12

**Authors:** Yanira Gonzalez, Baikuntha Aryal, Leena Chehab, V. Ashutosh Rao

**Affiliations:** Division of Therapeutic Proteins, Center for Drug Evaluation and Research, Food and Drug Administration

**Keywords:** autophagy, reactive oxygen species, mitoquinone, breast cancer

## Abstract

The interplay between oxidative stress and autophagy is critical for determining the fate of cancer cells exposed to redox-active and cytotoxic chemotherapeutic agents. Mitoquinone (MitoQ), a mitochondrially-targeted redox-active ubiquinone conjugate, selectively kills breast cancer cells over healthy mammary epithelial cells. We reported previously that MitoQ, although a derivative of the antioxidant ubiquinone, can generate excess ROS and trigger the Keap1-Nrf2 antioxidant response in the MDA-MB-231 cell line. Following MitoQ treatment, a greater number of cells underwent autophagy than apoptosis. However, the relationship between MitoQ-induced oxidative stress and autophagy as a primary cellular response was unclear. In this report, we demonstrate that MitoQ induces autophagy related gene 7 (Atg7)-dependent, yet Beclin-1-independent, autophagy marked by an increase in LC3-II. Both the *ATG7*-deficient human MDA-MB-231 cells and *Atg7*-knockout mouse embryonic fibroblasts exhibited lower levels of autophagy following MitoQ treatment than their respective wild-type counterparts. Increased apoptosis was confirmed in these autophagy-deficient isogenic cell line pairs, indicating that autophagy was attempted for survival in wild type cell lines. Furthermore, we observed higher levels of ROS in Atg7-deficient cells, as measured by hydroethidine oxidation. In Atg7-deficient cells, redox-sensitive Keap1 degradation was decreased, suggesting autophagy- and Atg7-dependent degradation of Keap1. Conversely, downregulation of Keap1 decreased autophagy levels, increased Nrf2 activation, upregulated cytoprotective antioxidant gene expression, and caused accumulation of p62, suggesting a feedback loop between ROS-regulated Keap1-Nrf2 and Atg7-regulated autophagy. Our data indicate that excessive ROS causes the upregulation of autophagy, and autophagy acts as an antioxidant feedback response triggered by cytotoxic levels of MitoQ.

## INTRODUCTION

Autophagy is a conserved lysosomal degradation pathway that ensures quality control of the cytoplasmic contents. In the normal cell, autophagy is present at low levels; damaged cellular organelles and protein aggregates are engulfed in autophagosomes to be degraded and recycled. However, under toxic stimuli, excessive autophagy can remove essential cellular components and may lead to cell death. During oxidative stress, when the amount of reactive oxygen species (ROS) exceeds the capacity of the antioxidant machinery, proteins, lipids, and DNA are oxidized and autophagy is induced [1]. Cancer cells have been reported to have higher levels of ROS than normal cells due to mitochondrial and metabolic dysfunction. While it might suppress tumor development, in these circumstances autophagy can support cancer progression by helping cells to survive in the stressful environment [2, 3].

Beclin-1 and its binding partner class III phosphoinositide 3-kinase (PI3K) are required for the formation of autophagosomes [4]. During autophagosome maturation, microtubule-associated protein light-chain 3 (LC3-I) is cleaved and then conjugated with phosphatidylethanolamine into LC3-II, a process mediated by the autophagy-related (Atg) proteins Atg7 and Atg3 [5]. Lipidated LC3-II is bound to the membrane of the autophagosome until fusion with the lysosome is complete. Thus, LC3-II acts as a biochemical marker for autophagy [6]. LC3-II is bound directly by the p62/SQSTM1 protein (hereafter referred to as p62), an autophagy substrate that serves as a cargo receptor for autophagic degradation of ubiquitinated and non-ubiquitinated substrate [7, 8]. Reduced levels of p62 can be used to confirm autophagic flux while accumulation of p62 is an indication of potential autophagy impairment [9]. Despite what is known about the mechanism of autophagy induction in normal cells, it remains unclear how Beclin-1, Atg7, and p62 signal for autophagy under excessive ROS conditions.

The Keap1–Nrf2 pathway senses free radicals and protects the cell during excessive oxidative and electrophilic conditions [10]. Keap1 regulates Nrf2 activity by binding and consequently mediating its polyubiquitination and subsequent proteosomal degradation under non-stressed conditions. Under stressful conditions, Nrf2 is released and accumulates in the nucleus where it binds to the antioxidant response element (ARE) sequence and induces the expression of cytoprotective antioxidant genes. In the absence of autophagy, p62 can accumulate and compete with Nrf2 to bind with Keap1. The association of p62 with Keap1 allows Nrf2 to activate antioxidant gene expression [11]. In addition, the *P62* gene contains the ARE promoter region, allowing Nrf2 to induce expression of *P62* under stressful conditions. This leads to a positive feedback loop where p62 activates Nrf2; constant Nrf2 activation stimulates *P62* expression under prolonged cellular stress. However, p62 is not required for initial activation of Nrf2 [12]. Keap1 mutation or p62 accumulation leads to a constitutively active Nrf2, which contributes to the growth of cancer cells [13–17]. Mutations that constitutively activate Nrf2 may contribute to tumorigenesis and chemoresistance in solid tumors [18]. Because absence of Keap1 leads to protein aggregation, it has been proposed that Keap1 plays a role in ubiquitin aggregate clearance via autophagy through association with LC3-II and p62 [19].

Furthermore, Keap1 accumulates in the absence of Atg7 or p62, indicating that autophagy and p62 are required for degradation of Keap1 [12, 20]. The p62 protein is phosphorylated during autophagy, which increases its affinity for Keap1 [17]. This increase in affinity causes the sequestration of Keap1 and p62 on autophagic cargos and their subsequent degradation [17, 21].

Excessive production of ROS via the mitochondrial electron transport chain can lead to oxidative damage if there is an imbalance between ROS and the antioxidant defense mechanism. Therefore, targeting an antioxidant directly to the mitochondria may decrease oxidative damage and protect the cells [22]. Our earlier work showed that Mitoquinone (MitoQ), a synthetic derivative of the antioxidant ubiquinone (CoQ10), induces oxidative stress and activates the Nrf2-Keap1 pathway [23, 24]. Although originally developed as an antioxidant, MitoQ has been reported to undergo redox cycling, produce ROS, and disrupt the inner mitochondrial membrane potential [23, 25]. Additionally, MitoQ exhibits selective anticancer activity and is more cytotoxic to cancer cells than healthy cells [23, 26]. In this report, we examined how Keap1 signals for an antioxidant response in the absence of autophagy and demonstrate that cross-talk between Atg7- and Keap1- dependent autophagy is a cell survival mechanism in response to MitoQ-induced oxidative stress.

## RESULTS

### MitoQ induces Beclin-1-independent and Atg7-dependent autophagy

**Figure 1:**
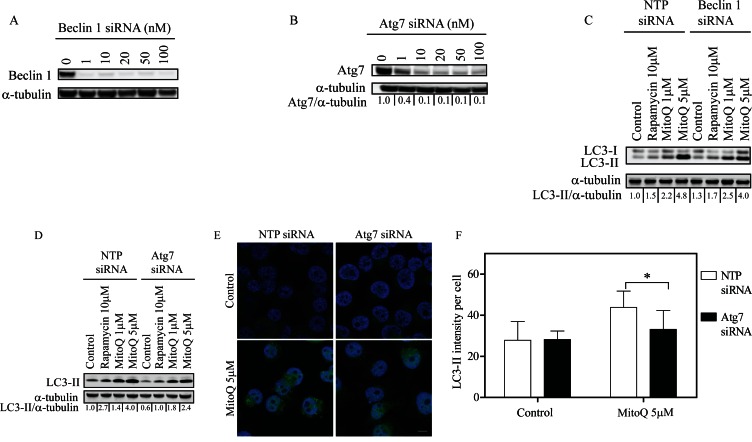
Knockdown of Atg7 inhibits MitoQ-induced autophagy in breast cancer cells. MDA-MB-231 cells were treated with increasing concentrations of siRNA oligonucleotides for 48 hr to downregulate Beclin-1 or Atg7. Cells were treated with **(A)** 10 nM Beclin-1 or **(B)** 10 nM Atg7 siRNA. Following 48 hr of **(C)** Beclin siRNA or **(D)** Atg7 siRNA treatment, cells were treated with MitoQ (1 or 5 μM) for 24 hr. Non-targeting pool (NTP) siRNA was used as a control. MitoQ-induced autophagy was measured using the levels of the autophagosome-associated LC3-II protein as an autophagy marker. Rapamycin (10 μM) was used as a positive control. **(E)** Representative confocal images and **(F)** LC3-II intensity per cell was used to confirm autophagy. n = 50 cells. Scale bar = 10 μm. Error bars represent S.D. *statistical significance compared with NTP siRNA cells (p<0.001).

To better understand the role of Beclin-1 or Atg7 in MDA-MB-231 cells exposed to MitoQ, we first optimized conditions for the downregulation of Beclin-1 or Atg7 protein using 10 nM siRNA oligonucleotides for 48 hr (Figure 1A and Figure 1B, respectively). We observed 90% knockdown of both proteins under these conditions. Non-targeting pool (NTP) siRNA oligonucleotides were used as controls. Cells transfected with control NTP siRNA as well as Beclin-1 siRNA showed an increase in LC3-II protein levels by Western blot following treatment with 1 μM or 5 μM MitoQ for 24 hr (Figure 1C). Downregulation of Beclin-1 in MDA-MB-231 cells had no significant effect upon the MitoQ-induced increase in autophagy. This indicates that MitoQ-induced autophagy is independent of Beclin-1 in MDA-MB-231 cells. However, in Atg7 siRNA-transfected cells, treatment with 5 μM MitoQ induced significantly lower levels of LC3-II compared to NTP siRNA transfected cells (Figure 1D). Rapamycin (10 μM) served as a positive control in each experiment. To confirm this observation, MDA-MB-231 cells were immunostained with anti-LC3-II antibody [27]. Consistent with Western blot analysis, cells treated with Atg7 siRNA showed lower LC3-II intensity per cell following treatment with 5 μM MitoQ for 24 hr (Figure 1E) as indicated by comparing the fluorescence intensities between control and Atg7 siRNA cells (Figure 1F).

To confirm that Atg7 is required for MitoQ-induced autophagy, we measured LC3-II levels in isogenic MEFs, wild type Atg7^+/+^ or Atg7^−/−^, exposed to either 1 μM or 5 μM MitoQ for 24 hr (Figure 2B). Following treatment with 5 μM MitoQ, MEF Atg7^+/+^ cells showed an increase in LC3-II with respect to control while LC3-II was not detectable in MEF Atg7^−/−^ control or treated cells. In particular, a higher concentration of MitoQ (5 μM) was required to induce autophagy in MEF Atg7^+/+^ than in breast cancer cells, which is consistent with previous observations [23, 26].

**Figure 2:**
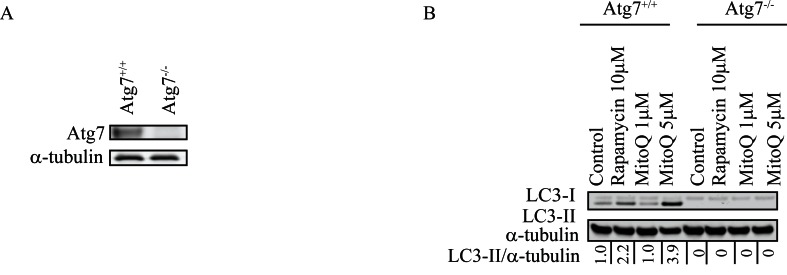
Knockout of Atg7 inhibits autophagy and increases apoptosis following MitoQ treatment in mouse embryonic fibroblasts **(A)** Levels of Atg7 were confirmed in wild type and Atg7^−/−^mouse embryonic fibroblast (MEF) cells. **(B)** Levels of LC3-II were used to compare autophagy induced by MitoQ (1 or 5 μM) following 24 hr of treatment in Atg7^+/+^ and Atg7^−/−^ MEF cells. Rapamycin (10 μM) was used as a positive control. **(C)** Annexin V and propidium iodide staining was used to measure induction of apoptosis by 5 μM MitoQ following 24 and 48 hr of treatment. Vp16 (1 μM) was used as a positive control. *statistical significance (p<0.05) compared with normalized control of Atg7^+/+^or Atg7^−/−^.

**Figure 2:**
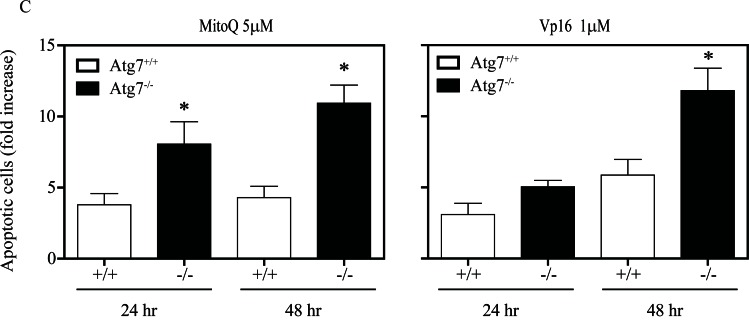
(C) Annexin V and propidium iodide staining was used to measure induction of apoptosis by 5 μM MitoQ following 24 and 48 hr of treatment. Vp16 (1 μM) was used as a positive control. *statistical significance (p<0.05) compared with normalized control of Atg7^+/+^or Atg7^−/−^.

To establish whether autophagy protects cells from MitoQ-induced cell death, we measured annexin V-PI positive staining. A significant increase in annexin V-PI positive staining was observed in Atg7^−/−^ MEF cells compared to Atg7^+/+^ MEF cells following 24 and 48 hr of MitoQ treatment (Figure 2C). Vp16 (10 μM), a positive control for apoptosis, also caused a significant increase in annexinV-PI positive staining in Atg7^−/−^ after 48 hr treatment. Lack of Atg7 enhanced MitoQ-induced apoptosis, which suggests that autophagy protects cells from MitoQ-induced cell death.

### Autophagy reduces MitoQ-induced ROS levels

**Figure 3:**
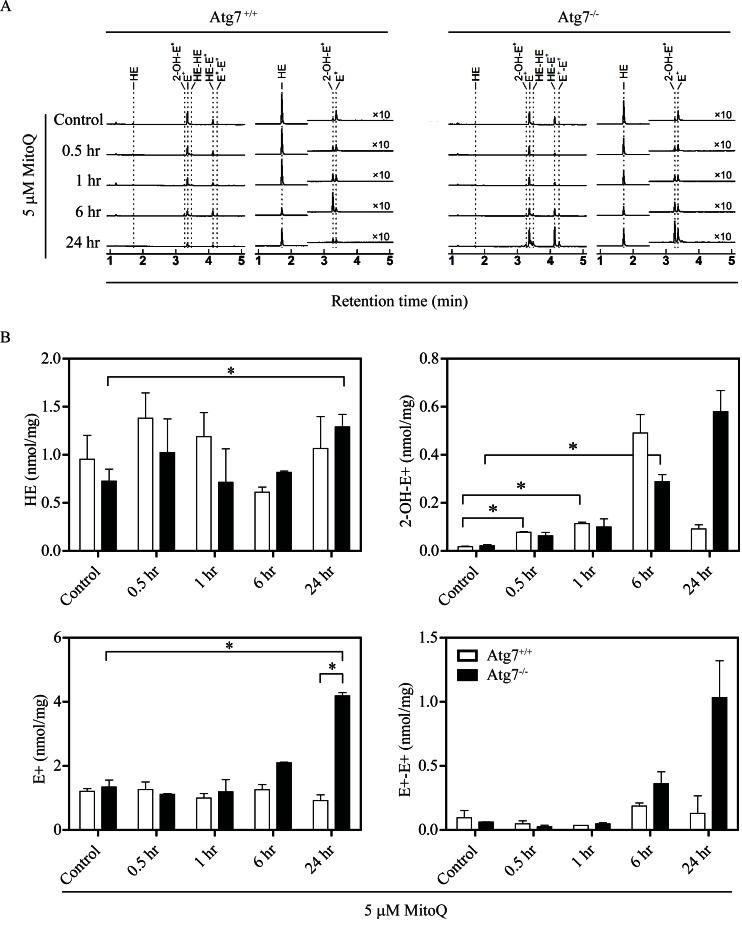
Autophagy-deficient cells have higher levels of ROS Cells were treated with 5 μM MitoQ for 0.5, 1, 6, or 24 hr. 10 μM hydroethidine (HE) was added for the last 0.5 hr of the MitoQ treatment. **(A)** Levels of ROS induced by MitoQ in MEF Atg7^+/+^or Atg7^−/−^ were measured by detection of HE and its oxidation products by HPLC (left traces: absorption signals at 290 nm, righ traces: fluorescence signals, with excitation/emission settings: 356 nm/440 nm for 0-2.5 min and 490 nm/ 567 nm for 2.5-6 min). **(B)** Graphs show quantitative analysis of HE, 2-OH-E^+^, E^+^, and E^+^-E^+^levels in cell lysates after normalization to protein concentration. Error bars represent S.D. *statistical significance (p<0.05).

To determine whether autophagy can modulate MitoQ-induced ROS, ROS production was measured in autophagy-deficient cells using an HPLC-based assay to identify hydroethidine (HE) oxidation products in autophagy deficient Atg7^−/−^ or wild type Atg7^+/+^ MEFs following treatment with 5 μM MitoQ for 0.5, 2, 6, or 24 hr (Figure 3A). Increase in HE oxidation product 2-hydroxyethidium (2-OH-E^+^) was statistically significant in Atg7^+/+^ cells following 0.5 and 1hr of MitoQ treatment, and in Atg7^−/−^ cells following 6hr of MitoQ treatment (Figure 3B). Statistically significant changes of ethidium (E^+^) were only observed in Atg7^−/−^ cells following 24 hr of MitoQ treatment. Moreover, 2-OH-E^+^, E^+^, and diethidium (E^+^-E^+^) were increased in autophagy-deficient cells to a greater extent than autophagy-proficient cells following treatment with 5 μM MitoQ for 24 hr. 2-OH-E^+^ levels were higher in Atg7^+/+^ following 6 hr of MitoQ treatment. However, statistically significant changes were only observed for E^+^ levels following 24 hr of 5 μM MitoQ treatment. The increased levels of 2-OH-E^+^, E^+^, and E^+^-E^+^ in autophagy-deficient cells after MitoQ treatment indicate an increase in one-electron oxidation of HE, possibly due to increased ROS formation, and that autophagy is required to decrease MitoQ-induced ROS. Due to increased level of diethidium, which is a specific marker of one-electron oxidation of the probe, the increased level of 2-OH-E^+^ may be interpreted not only in view of increased production of superoxide radical anion, but also increased one-electron oxidant(s), or both.

To determine the impact of autophagic signaling on oxidative stress-induced gene expression, we measured the expression levels of antioxidant genes in autophagy deficient Atg7^−/−^ or wild type Atg7^+/+^ MEFs. Deletion of *Atg7* induced downregulation of basal expression levels of cytoprotective genes, including heme oxygenase-1 (*Hmox1*), and peroxiredoxin 2 (*Prdx2*) (Supplemental Table 1). Conversely, treatment with 5 μM MitoQ for 24 hr upregulated the expression of the Nrf2-targeted genes, *Hmox1*, and *P62* in autophagy deficient Atg7^−/−^ and wild type Atg7^+/+^MEFs. Drug-induced upregulation of *Hmox1* and *P62* was higher in wild type cells than in autophagy deficient cells, indicative of an autophagic antioxidant role by triggering antioxidant gene expression. These data are also in agreement with the increased one-electron oxidation of hydroethidine in autophagy-deficient cells following MitoQ treatment (Figure 3B).

### Autophagy is required for Keap1 degradation following MitoQ treatment

**Figure 4:**
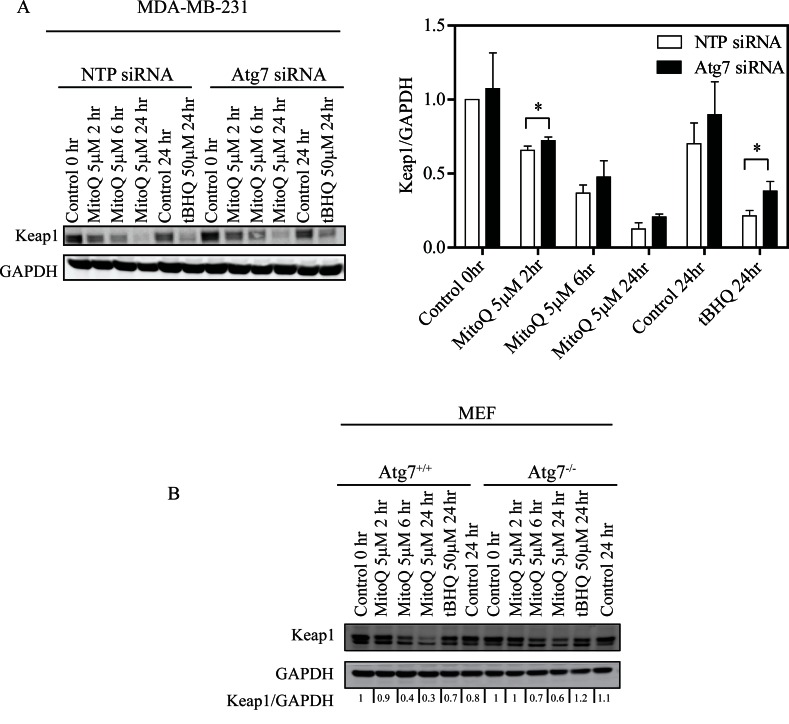
Depletion of Atg7 inhibits Keap1 degradation in breast cancer cells and MEF **(A)** MDA-MB-231 cells were transfected with 10 nM Atg7 siRNA or control NTP siRNA for 48 hr before MitoQ treatment. MDA-MB-231 cells were treated with 5 μM of MitoQ, and **(B)** Atg7^+/+^or Atg7^−/−^ MEF cells were treated with 5 μM MitoQ. Following 2, 6, or 24 hr of drug exposure, Keap1 degradation was analyzed by Western blot. 50μM tBHQ was used as a positive control. Error bars represent S.D. *statistical significance (p<0.05) compared with NTP siRNA cells.

To investigate whether autophagy/Atg7 is required for Keap1 degradation following MitoQ treatment, we measured Keap1 degradation by Western blot in an Atg7 deficient cell line. Keap1 is degraded in NTP and Atg7 knockdowns of MDA-MB-231 cells as well as in both Atg7^+/+^ and Atg7^−/−^ MEF cell lines following exposure to 5 μM MitoQ for 2, 6, or 24 hr (Figure 4A and Figure 4B). Keap1 degradation increases with exposure time to MitoQ. However, MitoQ-induced Keap1 degradation was reduced in MDA-MB-231 Atg7 siRNA following 2 hr treatment (Figure 4A). This effect was more apparent following treatment with 5 μM MitoQ than 1uM (data not shown) and in MDA-MB-231 cells than in MEFs. There was also a slight increase in the basal levels of Keap1 in Atg7 siRNA transfected MDA-MB-231 cell system. tBHQ (50 μM) was used as a positive control and also showed less Keap1 degradation in Atg7^−/−^ MEFs and Atg7 siRNA transfected MDA-MB-231 cells. Collectively, these data indicate that autophagy may be required for Keap1 degradation upon excessive ROS production.

### Depletion of Keap1 reduces MitoQ-induced autophagy

**Figure 5:**
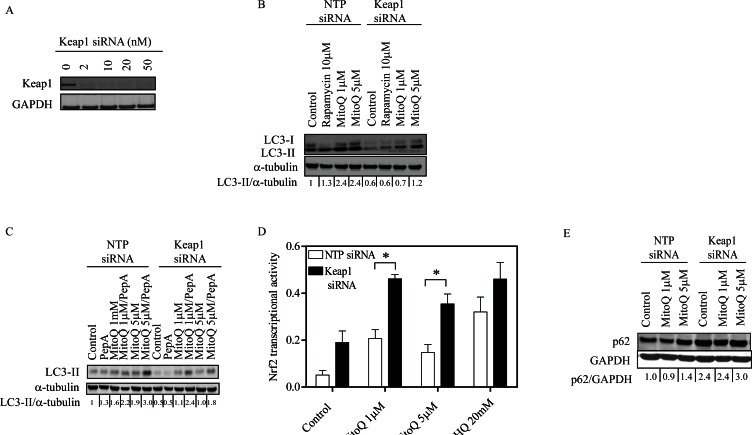
Depletion of Keap1 reduces MitoQ-induced autophagy and increases transcriptional activity of the antioxidant Nrf2 **(A)** MDA-MB-231 cells were treated with increasing concentrations of siRNA oligonucleotides for 24 hr to optimize the downregulation of Keap1. Cells were treated with 10 nM Keap1 siRNA or control NTP siRNA for 24 hr before being treated with MitoQ (1 or 5 μM) for 24 hr. **(B)** LC3-II protein was used as an autophagy marker. Rapamycin (10 μM) was used as a positive control. **(C)** Autophagic flux was determined by treating cells with the lysosomal protease inhibitors Pepstatin A (10 μg/ml) and E64d (10 μg/ml) in the presence or absence of MitoQ (1 or 5 μM) for 24 hr. **(D)** The transcriptional activity of Nrf2 was measured with an assay with immobilized oligonucleotide containing the ARE consensus binding site. tBHQ (20 μM) was used as a positive control. Error bars represent S.D. *statistical significance compared with NTP siRNA cells. **(E)** Autophagy impairment was measured by observing levels of the autophagy substrate p62.

To establish whether a feedback loop helps Keap1 regulate autophagy, we assayed LC3-II levels by Western blot in MDA-MB-231 cells with siRNA downregulation of Keap1. Optimal conditions for Keap1 knockdown were determined to be 10 nM siRNA oligonucleotides for 24 hr (Figure 5A). We observed MitoQ induced autophagy following 24 hr exposure; however, induction of autophagy was lower in Keap1 siRNA transfected cells compared to the control cells transfected with NTP siRNA (Figure 5B). Downregulation of Keap1 also decreased the basal levels of autophagy in MDA-MB-231 cells. These data suggest that in the absence of Keap1, autophagy response is diminished. To confirm that the increase in LC3-II following MitoQ treatment was indicative of autophagic flux, we inhibited the degradation of LC3-II by using pepstatin A and E64d [6]. Stabilization of LC3-II resulted in increased levels in NTP- and Keap1- siRNA transfected cells following MitoQ exposure (Figure 5C).

We then hypothesized that the difference in autophagy response observed in Keap1 deficient cells could be due to the fact that the antioxidant transcription factor Nrf2 is constitutively active, therefore excess free radicals are better scavenged and autophagy is not trigged. We analyzed the transcriptional activity of Nrf2 following MitoQ exposure. Basal levels of Nrf2 transcriptional activity increased in Keap1 siRNA- transfected cells, as did activity in NTP- and Keap1 siRNA- transfected cells following MitoQ exposure (Figure 5D). Significantly higher Nrf2 transcriptional activity was measured in Keap1 siRNA- than NTP siRNA- transfected cells following MitoQ treatment. To determine whether depletion of Keap1 had an effect on Nrf2-regulated gene expression, we measured the expression levels of antioxidant genes. Silencing Keap1 induced higher basal levels of Nrf2 regulated genes such as *HMOX1, NQO1,* and *P62* than from NTP siRNA (Supplemental Table 2). However, drug-induced upregulation of *HMOX1, NQO1,* and *P62* was diminished in response to MitoQ in Keap1 siRNA cells when compared to NTP siRNA cells.

As our data showed that the absence of Keap1 upregulates *P62* gene expression, we tested whether p62 protein level increases in the absence of Keap1. Downregulation of *Keap1* induced greater p62 accumulation, independent of MitoQ (Figure 5E). Together, these data showed that absence of Keap1 induces p62 protein accumulation.

## DISCUSSION

Autophagy can promote cell survival in response to stress [28]. In this study, we investigated the mechanism by which MitoQ induces autophagy via the cellular oxidative stress response. We demonstrated that: i) MitoQ induces autophagy independent of Beclin-1 and dependent on Atg7; ii) deletion of Atg7 increases ROS levels in response to MitoQ treatment; iii) Atg7 is required for Keap1 degradation; and iv) knockdown of Keap1 increases Nrf2 transcriptional activity, upregulates antioxidant genes expression, and decreases autophagy.

**Figure 6:**
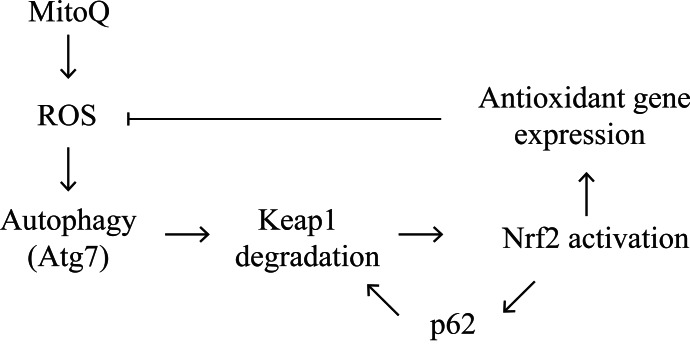
Schematic model for the proposed autophagy mechanism induced by MitoQ We propose a feedback autophagy and cellular response to excessive oxidative stress following cytotoxic exposure to MitoQ in cancer cells. Please refer to the discussion section for a detailed description.

Based on these findings, we propose that MitoQ increases ROS levels, resulting in induction of autophagy, which in turn exhibits a feedback antioxidant function (see model in Figure 6). MitoQ-induced ROS causes Keap1 oxidation followed by degradation via autophagy, and as a result, activation of Nrf2 [23, 29]. Nrf2 activation induces the expression of the antioxidant genes and *P62*. The p62 protein in turn associates with Keap1 and activates Nrf2, resulting in a positive feedback loop. We hypothesize that degradation of Keap1, the subsequent activation of Nrf2, and the expression of the antioxidant genes including p62, will decrease oxidative damage and consequently inhibit autophagy. It is possible that the induced levels of p62 gene expression by Keap1/Nrf2 are independent of Atg7-dependent autophagy because the levels of p62 expression were comparable in the Atg7 deficient and proficient cell lines.

Our data demonstrate that MitoQ-induced autophagy depends on Atg7 but not on Beclin-1. From previous studies it appears that Beclin-1-independent autophagy can occur in cancer cells as well as neurons following treatment with agents that induce cell death [30–32]. Therefore, a non-canonical pathway that does not require Beclin-1 or another redundant protein might be performing the same function to promote cell survival or cell death following drug treatment [33, 34]. As has been reported in hepatic cells [10], Atg7 is required for Keap1 degradation in two different model systems: MDA-MB-231 breast cancer cell lines and mouse embryonic fibroblasts. The kinetics of degradation was more prominent in MDA-MB-231 Atg7 siRNA cells than Atg7 knock-out MEFs. The reason for this might be the partial depletion of Atg7 using siRNA conditions versus an absence of Atg7 in knock-out cells. Additionally, Keap1 is almost completely degraded in MDA-MB-231 cells by 24 hr but not in MEFs, which might also be due to the presence of low levels of Atg7 in the siRNA MDA-MB-231 cells. These observations confirm that Keap1 is, at least partially, degraded via autophagy [29]. Moreover, we showed an increase in one-electron oxidation in autophagy deficient cells, which could be due to increase in superoxide and/or peroxidase-like activity [35, 36]. In addition, MitoQ decreased the ratio of reduced to oxidized (GSH/GSSG) glutathione in autophagy deficient cells, which demonstrates a more oxidized intracellular environment in the absence of autophagy (data not shown). Increase in cell death via apoptosis and upregulation of cytoprotective gene sin Atg7-deficient cells following MitoQ treatment indicates that autophagy is a cytoprotective response to rescue MEF cells from stress-induced death. Downregulation of basal levels of cytoprotective genes in the absence of autophagy/Atg7 demonstrated that expression of cytoprotective genes is dependent on MitoQ treatment/ROS. Moreover, higher expression of cytoprotective genes in the presence of autophagy confirmed that autophagy may be required to degrade Keap1, activate Nrf2, and express Nrf2-regulated genes. We demonstrate that lack of Keap1 decreases autophagy as well as increases Nrf2 transcriptional activity and antioxidant gene expression. In the absence of Keap1, basal expression levels of antioxidant genes are higher than the MitoQ-inducible levels, which are diminished. Previous work from our group suggested that Keap1 is a redox sensor and its oxidation targets it for degradation and subsequent Nrf2 activation [23]. The observed differences between basal levels and inducible levels of gene expression may be a consequence of Nrf2 being constitutively active. However it is also possible that another redox sensor is required to upregulate genes expression following MitoQ treatment. MitoQ-induced autophagy increases in the absence of Nrf2 [23]. We propose that autophagy decreases in the absence of Keap1 because there are lower levels of ROS following increased Nrf2 activation. Nrf2 could be activated either by degradation of Keap1 via autophagy, or by association of phosphorylated p62 with Keap1 [11, 15, 17, 37]. The accumulation of p62 when *Keap1* was downregulated is consistent with Nrf2-mediated *p62* upregulation and the positive feedback loop that they form [12]. However, it is also possible that Keap1 is required for p62 degradation because autophagosome formation is defective in *Keap1* knockout cells [19]. Our findings are also in concordance with previous reports relating the role of p62 in the regulation of Nrf2 activity and expression of *Nqo1* to maintain mitochondrial quality control [38]. Therefore, autophagy has an antioxidant role by degrading Keap1 to allow the release and activation of Nrf2 and the expression of cytoprotective antioxidant genes. It is likely that autophagy may be just one of different pathways involved in Keap1 degradation. Since Keap1degradation following MitoQ treatment was not completely abrogated in the absence of Atg7, it is possible that Keap1 is degraded by more than one pathway, including an Atg7-independent pathway [39].

Our present study demonstrates that MitoQ induces ROS, triggering the autophagy pathway which induces a cytoprotective antioxidant response. Treatment with an exogenous antioxidant (glutathione monoethyl ester) protected cells from MitoQ-induced autophagy (data not shown), confirming the role of excess ROS in signaling for autophagy. Previous studies have shown that ROS stimulates autophagy by modifying autophagy related proteins [40, 41]. Here we showed that ROS-induced autophagy degrades Keap1 and induces Nrf2 activation. The purpose of MitoQ-induced autophagy in cancer cells is to activate the Keap1/Nrf2 pathway and reduce oxidative stress. Further study is required to determine whether autophagy directly interferes with mitochondrial ROS production and other targets of free radical damage by eliminating damaged mitochondria through mitophagy. Further analysis is also necessary to understand the regulation of the Nrf2/Keap1 pathway in cancer treatment with autophagy inducers or inhibitors. Inhibition of autophagy would also lead to p62 accumulation and constitutive activation of Nrf2, offering tumor cells a defense against oxidative stress-inducing agents supporting the development of Nrf2 inhibitors as a therapeutic tool to enhance the function of chemotherapeutic drugs.

## MATERIALS AND METHODS

### Chemicals

MitoQ was synthesized as described previously [23]. *tert*-Butylhydroquinone (tBHQ), Etoposide (Vp-16), Rapamycin, Pepstatin A, and E64d were purchased from Sigma-Aldrich (St. Louis, MO).

### Chemicals

MDA-MB-231 breast cancer cells were obtained from ATCC (Manassas, VA). Cells were cultured in DMEM/F-12 medium containing 10% fetal bovine serum (FBS), 2 mM L-Glutamine, 1 mM sodium pyruvate (Mediatech, Manassas, VA). Mouse embryonic fibroblasts (MEFs) with Atg7^+/+^ and Atg7^−/−^genotypes were generously provided by Dr. Masaaki Komatsu (Tokyo Metropolitan Institute) and cultured in DMEM containing 10% FBS, 2 mM L-Glutamine, 1 mM sodium pyruvate. Cells were maintained at 37°C in 5% CO^2^.

### Knockdown of Beclin-1, Atg7, and Keap1

MDA-MB-231 cells were plated in growth media without antibiotics. After 24 hr, cells were transfected with ON-TARGETplus SMARTPOOL siRNA, 10 nM Beclin-1 (catalog no. L-010552-00), 10 nM Atg7 (catalog no. L-003755-00) or 10 nM Keap1 (catalog no. L-012453-00) using DharmaFECT 1 transfection reagent as described by the manufacturer (Thermo Scientific, Waltham, MA). ON-TARGETplus non-coding siRNA (catalog no. L-020112-00) served as a control in every experiment. Cells were transfected for 48 hr (for Beclin-1 siRNA or Atg7 siRNA) or 24 hr (for Keap1 siRNA) at 37°C in 5% CO^2^ before treating cells with drugs in fresh media.

### Western blotting

Cell and tissue lysate were prepared as described previously [23] and quantified by BCA assay. Total protein (~20-30 μg) was loaded and resolved in a Novex 4-12% Bis-Tris gel (Life Technologies, Carlsbad, CA). Protein was transferred to an Immobilon-P PVDF membrane (Millipore, Billerica, MA) and probed with anti-LC3-II (Novus Biologicals, Littleton, CO), anti-Beclin-1 (Novus Biologicals, Littleton, CO), anti-Atg7 (Sigma, St. Louis, MO), anti-p62 (BioLegend, San Diego, CA) or anti-Keap1 (Santa Cruz Biotechnology, Santa Cruz, CA) antibodies. Anti-ɑ–tubulin (Cell Signaling Technology, Danvers, MA) or anti-GAPDH (Imgenex, San Diego, CA) were used as loading controls. Protein levels were quantified using densitometric scanning and normalized to loading control levels.

### Immunohistochemistry

MDA-MB-231 cells were plated and transfected with siRNA on chamber slides (Thermo Fisher Scientific, Rockville, MD). Following drug treatment, cells were fixed with 4% paraformaldehyde in PBS and subsequently permeabilized with 100 μg/mL digitonin in PBS. Cell were treated with 1% BSA and incubated with anti-LC3-II (MBL, Woburn, MA), followed by secondary antibody conjugated with Alexa dyes (Life Technologies, Carlsbad, CA). Slides were mounted with cover-slips with DAPI-containing mounting solution (Vector Laboratories, Burlingame, CA). Digital images were acquired with a Zeiss LSM 700 confocal system using a 63X oil-immersion objective.

### Annexin-V staining

For apoptosis analysis, post-treatment and control MEF cells were collected and stained using an Annexin V-FITC Apoptosis Detection Kit II (BD Pharmingen, San Jose, CA) as described by the manufacturer. Stained cells were analyzed by a FACSCaliber flow cytometer (BD Biosciences, San Jose, CA).

### Hydroethidine (HE) assay with HPLC analysis

To detect ROS following MitoQ treatment, MEF (Atg7^+/+^ or Atg7^−/−^) cells were treated with 5 μM MitoQ for 0.5, 1, 6, and 24 hr. During the last 0.5 hr of MitoQ treatment, cells were co-treated with 10 μM hydroethidine (HE). The cells were processed and analyzed by HPLC with absorption and fluorescence detection to identify HE oxidation products as described previously [42] with subsequent modifications [43].

### Transcriptional activity of Nrf2

An ELISA-based assay was performed to measure the DNA binding activity of Nrf2 (TransAM Kit, Active Motif, catalog no. 50296) in MDA-MB-231 cell extracts as reported previously [23].

### Oxidative stress RT^2^ profiler PCR array RNA Isolation

MDA-MB-231 cells (NTP siRNA or Keap1 siRNA) and Atg7^+/+^ or Atg7^−/−^ MEF cells were treated with 5 μM MitoQ for 24 hr. Cell pellets were frozen and shipped to Super-Array Bioscience (Frederick, MD) for gene expression analysis. RNA was isolated using the RNEasy Mini Kit (QIAGEN, Valencia, CA catalog no. 74104) following the manufacturer's protocol. RNA quality was determined using the Agilent Bioanalyzer (Agilent, Santa Clara, CA) with RNA 6000 Nano Kits (Agilent, catalog no. 5067-1511). Total RNA yield, 260/280, and 260/230 ratios were measured using a NanoDrop spectrophotometer (Thermo Scientific, Waltham, MA).

### Quantitative RT-PCR

Reverse Transcription reaction was performed using 500 ng RNA input per sample to analyze the expression of 84 genes related to oxidative stress. RT^2^ Profiler™ PCR Array Human Oxidative Stress (Super-Array Bioscience, Frederick, MD) was used to analyze gene expression in MDA-MB-231 cells (NTP siRNA or Keap1 siRNA), and RT^2^ Profiler™ PCR Array Mouse Oxidative Stress (Super-Array Bioscience, Frederick, MD) was used to determine gene expression in Atg7^+/+^ and Atg7^−/−^ MEF cells. RT-PCR was performed using the Applied Biosystem 7900 (Life Technologies, Calsbad, CA) in 384-well format. Ct values were normalized using the average Ct value of ACTB, B2M, GAPDH, HPRT1, and RPLP0. Data analysis was performed using the data analysis tool which can be found at http://pcrdataanalysis.sabiosciences.com/pcr/arrayanalysis.php.

### Statistics

Western blot quantification, annexin-V/PI fluorescence, HE oxidation products, and Nrf2 transcriptional activity were compared and analyzed by Student's *t*-test. A value of *p* ≤ 0.05 was considered statistically significant.

## SUPPLEMENTARY TABLES



## References

[1] Li L, Ishdorj G, Gibson SB (2012). Reactive oxygen species regulation of autophagy in cancer: implications for cancer treatment. Free Radic Biol Med.

[2] Kimmelman AC (2011). The dynamic nature of autophagy in cancer. Genes Dev.

[3] Yue Z, Jin S, Yang C, Levine AJ, Heintz N (2003). Beclin 1, an autophagy gene essential for early embryonic development, is a haploinsufficient tumor suppressor. Proceedings of the National Academy of Sciences of the United States of America.

[4] Amaravadi RK, Lippincott-Schwartz J, Yin XM, Weiss WA, Takebe N, Timmer W, DiPaola RS, Lotze MT, White E (2011). Principles and current strategies for targeting autophagy for cancer treatment. Clin Cancer Res.

[5] Taherbhoy AM, Tait SW, Kaiser SE, Williams AH, Deng A, Nourse A, Hammel M, Kurinov I, Rock CO, Green DR, Schulman BA (2011). Atg8 transfer from Atg7 to Atg3: a distinctive E1-E2 architecture and mechanism in the autophagy pathway. Mol Cell.

[6] Klionsky DJ, Abdalla FC, Abeliovich H, Abraham RT, Acevedo-Arozena A, Adeli K, Agholme L, Agnello M, Agostinis P, Aguirre-Ghiso JA, Ahn HJ, Ait-Mohamed O, Ait-Si-Ali S, Akematsu T, Akira S, Al-Younes HM (2012). Guidelines for the use and interpretation of assays for monitoring autophagy. Autophagy.

[7] Nezis IP, Stenmark H (2012). p62 at the interface of autophagy, oxidative stress signaling, and cancer. Antioxid Redox Signal.

[8] Pankiv S, Clausen TH, Lamark T, Brech A, Bruun JA, Outzen H, Overvatn A, Bjorkoy G, Johansen T (2007). p62/SQSTM1 binds directly to Atg8/LC3 to facilitate degradation of ubiquitinated protein aggregates by autophagy. The Journal of biological chemistry.

[9] Bjorkoy G, Lamark T, Brech A, Outzen H, Perander M, Overvatn A, Stenmark H, Johansen T (2005). p62/SQSTM1 forms protein aggregates degraded by autophagy and has a protective effect on huntingtin-induced cell death. J Cell Biol.

[10] Taguchi K, Motohashi H, Yamamoto M (2011). Molecular mechanisms of the Keap1-Nrf2 pathway in stress response and cancer evolution. Genes Cells.

[11] Komatsu M, Kurokawa H, Waguri S, Taguchi K, Kobayashi A, Ichimura Y, Sou YS, Ueno I, Sakamoto A, Tong KI, Kim M, Nishito Y, Iemura S, Natsume T, Ueno T, Kominami E (2010). The selective autophagy substrate p62 activates the stress responsive transcription factor Nrf2 through inactivation of Keap1. Nat Cell Biol.

[12] Jain A, Lamark T, Sjottem E, Larsen KB, Awuh JA, Overvatn A, McMahon M, Hayes JD, Johansen T (2010). p62/SQSTM1 is a target gene for transcription factor NRF2 and creates a positive feedback loop by inducing antioxidant response element-driven gene transcription. The Journal of biological chemistry.

[13] Hayes JD, McMahon M (2009). NRF2 and KEAP1 mutations: permanent activation of an adaptive response in cancer. Trends Biochem Sci.

[14] Moscat J, Diaz-Meco MT (2009). p62 at the crossroads of autophagy, apoptosis, and cancer. Cell.

[15] Inami Y, Waguri S, Sakamoto A, Kouno T, Nakada K, Hino O, Watanabe S, Ando J, Iwadate M, Yamamoto M, Lee MS, Tanaka K, Komatsu M (2011). Persistent activation of Nrf2 through p62 in hepatocellular carcinoma cells. J Cell Biol.

[16] Komatsu M, Kageyama S, Ichimura Y (2012). p62/SQSTM1/A170: physiology and pathology. Pharmacol Res.

[17] Ichimura Y, Waguri S, Sou YS, Kageyama S, Hasegawa J, Ishimura R, Saito T, Yang Y, Kouno T, Fukutomi T, Hoshii T, Hirao A, Takagi K, Mizushima T, Motohashi H, Lee MS (2013). Phosphorylation of p62 activates the Keap1-Nrf2 pathway during selective autophagy. Mol Cell.

[18] Ganan-Gomez I, Wei Y, Yang H, Boyano-Adanez MC, Garcia-Manero G (2013). Oncogenic functions of the transcription factor Nrf2. Free radical biology & medicine.

[19] Fan W, Tang Z, Chen D, Moughon D, Ding X, Chen S, Zhu M, Zhong Q (2010). Keap1 facilitates p62-mediated ubiquitin aggregate clearance via autophagy. Autophagy.

[20] Guo W, Kan JT, Cheng ZY, Chen JF, Shen YQ, Xu J, Wu D, Zhu YZ (2012). Hydrogen sulfide as an endogenous modulator in mitochondria and mitochondria dysfunction. Oxid Med Cell Longev.

[21] Bae SH, Sung SH, Oh SY, Lim JM, Lee SK, Park YN, Lee HE, Kang D, Rhee SG (2013). Sestrins activate Nrf2 by promoting p62-dependent autophagic degradation of Keap1 and prevent oxidative liver damage. Cell metabolism.

[22] Murphy MP (1997). Selective targeting of bioactive compounds to mitochondria. Trends Biotechnol.

[23] Rao VA, Klein SR, Bonar SJ, Zielonka J, Mizuno N, Dickey JS, Keller PW, Joseph J, Kalyanaraman B, Shacter E (2010). The antioxidant transcription factor Nrf2 negatively regulates autophagy and growth arrest induced by the anticancer redox agent mitoquinone. The Journal of biological chemistry.

[24] Kelso GF, Porteous CM, Coulter CV, Hughes G, Porteous WK, Ledgerwood EC, Smith RA, Murphy MP (2001). Selective targeting of a redox-active ubiquinone to mitochondria within cells: antioxidant and antiapoptotic properties. The Journal of biological chemistry.

[25] Doughan AK, Dikalov SI (2007). Mitochondrial redox cycling of mitoquinone leads to superoxide production and cellular apoptosis. Antioxid Redox Signal.

[26] Cheng G, Zielonka J, Dranka BP, McAllister D, Mackinnon AC, Joseph J, Kalyanaraman B (2012). Mitochondria-targeted drugs synergize with 2-deoxyglucose to trigger breast cancer cell death. Cancer Res.

[27] Fujita K, Maeda D, Xiao Q, Srinivasula SM (2011). Nrf2-mediated induction of p62 controls Toll-like receptor-4-driven aggresome-like induced structure formation and autophagic degradation. Proceedings of the National Academy of Sciences of the United States of America.

[28] Mehrpour M, Esclatine A, Beau I, Codogno P (2010). Autophagy in health and disease. 1. Regulation and significance of autophagy: an overview. Am J Physiol Cell Physiol.

[29] Taguchi K, Fujikawa N, Komatsu M, Ishii T, Unno M, Akaike T, Motohashi H, Yamamoto M (2012). Keap1 degradation by autophagy for the maintenance of redox homeostasis. Proceedings of the National Academy of Sciences of the United States of America.

[30] Scarlatti F, Maffei R, Beau I, Codogno P, Ghidoni R (2008). Role of non-canonical Beclin 1-independent autophagy in cell death induced by resveratrol in human breast cancer cells. Cell Death Differ.

[31] Grishchuk Y, Ginet V, Truttmann AC, Clarke PG, Puyal J (2011). Beclin 1-independent autophagy contributes to apoptosis in cortical neurons. Autophagy.

[32] Smith DM, Patel S, Raffoul F, Haller E, Mills GB, Nanjundan M (2010). Arsenic trioxide induces a beclin-1-independent autophagic pathway via modulation of SnoN/SkiL expression in ovarian carcinoma cells. Cell Death Differ.

[33] Codogno P, Mehrpour M, Proikas-Cezanne T (2012). Canonical and non-canonical autophagy: variations on a common theme of self-eating?. Nat Rev Mol Cell Biol.

[34] Juenemann K, Reits EA (2012). Alternative macroautophagic pathways. Int J Cell Biol.

[35] Pua HH, Guo J, Komatsu M, He YW (2009). Autophagy is essential for mitochondrial clearance in mature T lymphocytes. Journal of immunology.

[36] Wu JJ, Quijano C, Chen E, Liu H, Cao L, Fergusson MM, Rovira II, Gutkind S, Daniels MP, Komatsu M, Finkel T (2009). Mitochondrial dysfunction and oxidative stress mediate the physiological impairment induced by the disruption of autophagy. Aging.

[37] Lau A, Wang XJ, Zhao F, Villeneuve NF, Wu T, Jiang T, Sun Z, White E, Zhang DD (2010). A noncanonical mechanism of Nrf2 activation by autophagy deficiency: direct interaction between Keap1 and p62. Mol Cell Biol.

[38] Kwon J, Han E, Bui CB, Shin W, Lee J, Lee S, Choi YB, Lee AH, Lee KH, Park C, Obin MS, Park SK, Seo YJ, Oh GT, Lee HW, Shin J (2012). Assurance of mitochondrial integrity and mammalian longevity by the p62-Keap1-Nrf2-Nqo1 cascade. EMBO Rep.

[39] Zhang DD, Lo SC, Sun Z, Habib GM, Lieberman MW, Hannink M (2005). Ubiquitination of Keap1, a BTB-Kelch substrate adaptor protein for Cul3, targets Keap1 for degradation by a proteasome-independent pathway. The Journal of biological chemistry.

[40] Lee J, Giordano S, Zhang J (2012). Autophagy, mitochondria and oxidative stress: cross-talk and redox signalling. The Biochemical journal.

[41] Scherz-Shouval R, Shvets E, Fass E, Shorer H, Gil L, Elazar Z (2007). Reactive oxygen species are essential for autophagy and specifically regulate the activity of Atg4. The EMBO journal.

[42] Zielonka J, Vasquez-Vivar J, Kalyanaraman B (2008). Detection of 2-hydroxyethidium in cellular systems: a unique marker product of superoxide and hydroethidine. Nat Protoc.

[43] Zielonka J, Zielonka M, Sikora A, Adamus J, Joseph J, Hardy M, Ouari O, Dranka BP, Kalyanaraman B (2012). Global profiling of reactive oxygen and nitrogen species in biological systems: high-throughput real-time analyses. The Journal of biological chemistry.

