# Supplementary material for: Atg7- and Keap1-dependent autophagy protects breast cancer cell lines against mitoquinone-induced oxidative stress
Source: Oncotarget. 2014 Mar 12;5(6):1526–37. doi: 10.18632/oncotarget.1715 (PMC4039229; doi:10.18632/oncotarget.1715)
Supplement: Supplementary file 1 [file oncotarget-05-1526-s001.pdf]

Supplementary Table S1: Regulation of antioxidant gene expression following MitoQ treatment in mouse embryonic fibroblasts.

Gene name	Symbol	$Atg7^{+/+}$ MEF MitoQ-treated[a]	$Atg7^{-/-}$ MEF MitoQ-treated[b]	$Atg7^{-/-}$ MEF Basal levels[c]
Chemokine (C-C motif) ligand 5	*Ccl5*	2.9773	2.7587	1.2816
EH-domain containing 2	*Ehd2*	−2.8206	−1.554	−1.1583
Heme oxygenase (decycling) 1	*Hmox1*	17.196	11.6641	−5.2488
Heat shock protein 1A	*Hspa1a*	9.5401	1.7219	16.5413
Peroxiredoxin 1	*Prdx1*	−1.9132	−2.5245	−1.2588
Peroxiredoxin 2	*Prdx2*	−1.3435	−1.3717	−2.9526
Prion protein	*Prnp*	2.0763	2.6833	−1.9079
Prostaglandin-endoperoxide synthase 1	*Ptgs1*	1.9779	−1.3623	−3.6351
Prostaglandin-endoperoxide synthase 2	*Ptgs2*	7.9118	8.5386	1.0483
RecQ protein-like 4	*Recql4*	2.9567	2.521	−1.3775
Superoxide dismutase 3, extracellular	*Sod3*	1.9106	−2.0364	1.3736
Sequestosome 1	*P62/Sqstm1*	4.8032	3.9014	1.0196
Thioredoxin interacting protein	*Txnip*	−6.8022	−9.038	2.425

[a]Fold change following 5 µM MitoQ treatment in wild type cells with respect to non-treated control.
[b]Fold change following 5 µM MitoQ treatment in *Atg7* knockout cells with respect to non-treated control.
[c]Basal level of gene expression in *Atg7* knockout cells with respect to basal level of wild type (=1).

Supplementary Table S2: Regulation of antioxidant gene expression following MitoQ treatment in MDA-MB-231 cells depleted of Keap1.

Gene name	Symbol	NTP siRNA MitoQ-treated[a]	Keap1 siRNA MitoQ-treated[b]	Keap1 siRNA Basal levels[c]
24-dehydrocholesterol reductase	*DHCR24*	−2.3362	−2.0513	−1.1846
Dual oxidase 1	*DUOX1*	1.4299	2.5428	−1.5436
Dual specificity phosphatase 1	*DUSP1*	4.9088	4.5751	1.2432
Forkhead box M1	*FOXM1*	−4.3768	−3.9884	1.2452
Ferritin, heavy polypeptide 1	*FTH1*	8.8441	4.113	2.8805
Glutamate-cysteine ligase, catalytic subunit	*GCLC*	4.0941	1.283	3.4871
Glutamate-cysteine ligase, modifier subunit	*GCLM*	4.0367	1.4533	3.8816
Glutathione peroxidase 3 (plasma)	*GPX3*	−2.3113	−4.2539	1.8415
Glutathione reductase	*GSR*	2.145	−1.0499	2.7267
Glutathione S-transferase pi 1	*GSTP1*	1.2822	3.4292	−2.2642
Heme oxygenase (decycling) 1	*HMOX1*	8.8221	2.5077	7.3908
Neutrophil cytosolic factor 2	*NCF2*	16.858	6.7034	4.6693
NAD(P)H dehydrogenase, quinone 1	*NQO1*	2.5671	1.2212	2.798
Nudix-type motif 1	*NUDT1*	−1.9038	−2.8535	1.2183
Peroxiredoxin 5	*PRDX5*	2.3782	2.2512	1.2074
Prion protein	*PRNP*	2.8399	2.3299	−1.015
Prostaglandin-endoperoxide synthase 2	*PTGS2*	8.8226	15.9656	−1.202
Ring finger protein 7	*RNF7*	2.2849	2.0984	1.0319
Scavenger receptor class A, member 3	*SCARA3*	−2.5788	−2.953	−1.3939
Selenoprotein S	*SELS*	2.4118	2.0514	−1.0724
Superoxide dismutase 3, extracellular	*SOD3*	−2.2526	−1.6342	−1.3068
Sequestosome 1	*P62/SQSTM1*	13.091	5.568	2.9663
Sulfiredoxin 1	*SRXN1*	27.8443	7.8913	5.4315
Thioredoxin	*TXN*	2.1904	1.3914	2.0195
Thioredoxin reductase 1	*TXNRD1*	6.6545	2.0075	2.7461
Uncoupling protein 2	*UCP2*	−2.1402	−2.5191	−1.0277

[a]Fold change following 5 μM MitoQ treatment in control cells with NTP siRNA with respect to non-treated control.
[b]Fold change following 5 μM MitoQ treatment in cells with knocked-down Keap1 with respect to non-treated control.
[c]Basal level of gene expression in cells depleted of Keap1 with respect to basal level of NTP siRNA cells (=1).
